# Active electrochemical high-contrast gratings as on/off switchable and color tunable pixels

**DOI:** 10.1038/s41467-022-31083-z

**Published:** 2022-06-13

**Authors:** Cheon Woo Moon, Youngji Kim, Jerome Kartham Hyun

**Affiliations:** Department of Chemistry and Nanoscience, Ewha Womans University, Seoul, 03760 Republic of Korea

**Keywords:** Nanocavities, Nanophotonics and plasmonics, Electrochemistry

## Abstract

To be viable for display applications, active structural colors must be electrically tunable, on/off switchable, and reversible. Independently controlling the first two functions, however, is difficult because of causality that ties the real and imaginary parts of the optical constants or changing overlap of fields during structural variations. Here, we demonstrate an active reflective color pixel that encompasses separate mechanisms to achieve both functions reversibly by electrochemically depositing and dissolving Cu inside the dielectric grating slits on a Pt electrode with ΔV < 3 V. Varying the modal interference via Cu occupancy in the slits changes the CIE space coverage by up to ~72% under cross-polarized imaging. In the same pixel, depolarization and absorption by the dissolving porous Cu switches the color off with a maximum contrast of ~97%. Exploiting these results, we demonstrate an active color-switching display and individually addressable on/off pixel matrix that highlights their potential in reflective display applications.

## Abstract (web summary)

Two key display operations, color tuning and on/off switching, are achieved with reflective structural colors by changing the modal interference conditions and absorption via electrochemical control of Cu occupancy inside dielectric grating slits.

## Introduction

Ideally, electrically tunable reflective pixels for display applications^1,2^ or optically variable devices (OVDs)^3^ should change colors over a wide range upon applied bias, switch off when needed, exhibit high contrast against the background, and remain reversible. Controlling the first two criteria with a single structural color design is particularly challenging because they require seemingly distinct mechanisms. Active color tuning requires a change in the real part of the optical constants or an ordered structural variation, whereas on/off switching demands a change in the imaginary part or the introduction of structural disorder to increase and broaden the absorption. Because the real and imaginary parts of the optical constant are tied by causality (i.e., Kramers–Kronig relation) and structural variations affect the coupling between fields, color tuning is inevitably accompanied by intensity modulations, challenging independent control of color and intensity. This suggests that color tuning and on/off switching should rely on separate mechanisms, which are not straightforward to encompass in one system. Studies focused on one or the other have relied on electromechanical Mie structures^4^ and plasmonic or photonic designs paired with electrochromic polymers^5–8^, liquid crystals^9,10^, metal electrodeposition^11–13^, metal-to-dielectric electrochemical conversion^14,15^, metal hydrogenation^16^, and ion intercalation^17–19^.

Prolonged reversibility is another important requirement. While plasmonic colors offer attractive merits^20,21^, the workhorse metals, namely Ag and Al, are easily degraded, compromising the color stability with repeated use. Other metals with superior chemical stability such as Pt are generally avoided because of large optical damping factors associated with interband transitions^22^. Nonetheless, vibrant structural colors, largely insensitive to the optical loss of the metal, are achievable using a 1-dimensional (1D) dielectric grating on the respective metal, rotated 45° against the input polarization and observed through a cross (orthogonal) polarizer^23^. Vibrant structural colors from such a cross-polarized scheme have also been reported for plasmonic nanowires^24^ and dielectric nanopixels on Ag^25^. For the dielectric 1D grating, the color is defined by the reflected field rotated 90° across the s-polarized (s-pol) or p-polarized (p-pol) resonance wavelengths. The p-pol resonance, described by the surface plasmon polariton (SPP) at the metal and grating interface, is attenuated for stable but lossy metals like Pt. This leaves the s-pol resonance, generally characterized by the Rayleigh–Wood anomaly, to dominate the reflection spectrum resulting in highly saturated colors owing to the sharp nature of its peak, as has been observed in previous reports^26,27^.

Herein, we report on electrochemically controllable structural colors that display active and passive color tunability, on/off switching, high contrast against the background, and good chemical stability. We employ a TiO_x_ grating on a Pt electrode in the configuration described above, where the optical response is modulated via redox of Cu ions on the exposed parts of the Pt in an electrolyte and the electrochemical stability is ensured by Pt. Unlike plasmonic or electrochromic designs, our system achieves active color tuning by electrically converting the modal interference via Cu occupancy inside the grating slits, providing intensity unencumbered by plasmonic loss. On the other hand, on/off switching is realized from the same design via increased plasmonic loss and scrambled scattering by the disordered porous Cu formed during dissolution. Our approach enables both color tuning and on/off switching, necessary for implementing structural colors in active reflective displays.

## Results and discussion

### Active color tuning mechanism

**Fig. 1:**
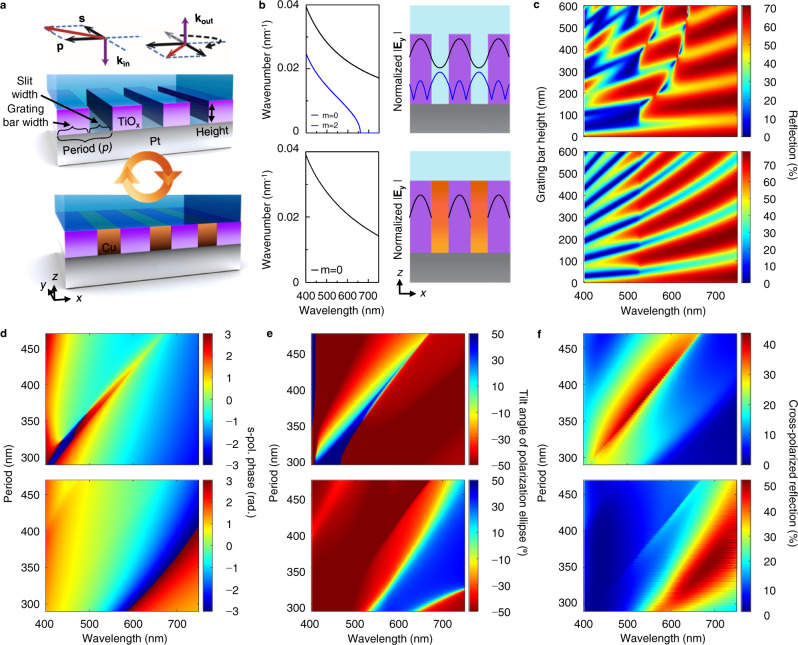
Shift in optical response between bare and Cu-filled HCG under s-pol light. **a** Schematic of a bare and Cu-filled HCG normally excited by the superposition of s- and p-pol light. The reflected electric field vector is rotated via phase shifts to the s- and/or p-pol field. **b** Dispersion curves of waveguide-array modes (left) and their modal profiles at the wavelength of 550 nm (right) for the bare (top) and Cu-filled HCG (bottom). **c** Calculated 0th order reflection of bare (top) and Cu-filled HCG (bottom) as a function of grating bar height and wavelength. **d**–**f** Phase of the 0th order s-pol reflected field (**d**), tilt angle of polarization ellipse (**e**), and cross-polarized reflection (**f**) for the bare (top) and Cu-filled HCG (bottom) as a function of period and wavelength.

Our high-contrast gratings (HCGs) achieve color tunability by changing the wavelength-dependent birefringence via electrodeposited Cu inside the grating (Fig. 1a). An HCG whose period (p) lies between the wavelength in the grating bar and the slits can support more than one mode that travels between the top and bottom interface. These modes are known as Bloch waves^28^ or waveguide-array modes^29^, different from the laterally propagating waves of guided-mode resonances (GMRs)^30^, although both types can explain the same grating phenomena. It is the propagation and interplay of the waveguide-array modes that determine the overall reflection characteristics of the HCG^29^. To illustrate this point, we analytically calculate the waveguide-array modes of a TiO_x_ grating with *p* = 370 nm and a slit width of 165 nm in a dielectric environment of *n* = 1.42 excited by normally incident light (see Methods and Supplementary Note 1). Since the p-pol excitation induces a plasmonic response that is swiftly attenuated by the lossy Pt, we focus on the s-pol response of the grating. Two propagating wavevectors, *β*_0_ and *β*_2_, are found between *λ* = 400 and 660 nm with field intensities maximized inside the HCG bars and slits, respectively (Fig. 1b, top). For a HCG on a Pt substrate, the phase accumulation and subsequent interference of these two modes over varying grating heights lead to a complex pattern in the 0th order reflection spectra (Fig. 1c, top).

We now show that this complex behavior can be dramatically simplified with a Cu-filled HCG. Unlike dielectrics, metals tend to reject all fields within their structure, especially at long wavelengths. Therefore, the mode, *β*_2_, whose field was previously maximized inside the empty slits, cannot be excited, whereas the fundamental mode, *β*_0_, remains because it propagates inside the grating bar. Indeed, analytical calculations using surface impedance boundary conditions^31,32^ (see Methods and Supplementary Note 1) show that only *β*_0_ exists in a Cu-filled HCG (Fig. 1b, bottom) whose modal profile and dispersion are similar to those of the bare HCG *β*_0_. Being governed by a single mode, the reflection spectra resemble the simple behavior of a homogeneous thin film, with reflection bands repeating over grating bar height (Fig. 1c, bottom).

The s-pol response for both bare and Cu-filled HCGs can be better understood by analyzing the phase spectra of the 0th order reflected field over the HCG period. For the bare HCG with a height of 100 nm, a ~π phase shift occurs with the Rayleigh–Wood anomaly (Fig. 1d, top). When the empty slits are filled by Cu, the resonance redshifts by ~135 to ~200 nm over the range of considered periods (Fig. 1d, bottom) since the number of resonance field anti-nodes in the HCG unit cell is reduced (Supplementary Fig. 1). Unlike most structural color designs paired with electrochromic polymers or electrodeposition schemes, this shift is not determined by refractive index variations nor changes to the field overlap, but rather the ‘muting’ of resonance fields inside the HCG slit and the subsequent change in modal interference. We note that as a consequence of this mechanism, the colors are dependent on the incident angle since off-normal incidence introduces odd modes (i.e., *β*_1_, *β*_3_, etc) that modify the interference conditions.

For an HCG rotated −45° against the incident polarization and observed through a cross-polarizer (Fig. 1a), the s-pol resonances for both bare and Cu-filled HCGs are directly associated with large reflection intensities as the tilt angle of the polarization ellipse is close to 45° at the resonances and −45° otherwise (Fig. 1e). This results because the p-pol resonance is attenuated by the lossy metals, leaving the s-pol resonance alone to determine the tilt angle. The scheme offers a useful benefit for color generation. For both bare and Cu-filled HCGs, the s-pol resonance is not as strongly affected by the metallic loss as the p-pol response since its field maxima are distant from the metal surface. As a result, the cross-polarized reflection intensities remain strong for both HCGs (Fig. 1f). We note that, unlike the bare and Cu-filled HCGs, partially Cu-filled HCGs rely on both s and p-pol responses to generate the cross-polarized reflection (see Supplementary Note 2 and Supplementary Fig. 2), leading to a cross-polarized response much broader than the s-pol response alone. This is attributed to the interference of the fundamental p-pol mode within the bare part of the HCG, also yielding a ~π phase shift across the wavelengths.

### Operation of electrochemical structural color pixel

To experimentally observe the spectral shifts, we configured our imaging setup according to the scheme of Fig. 1a, with details shown in Supplementary Fig.  3. Polarized white light was directed and focused onto the active color pixel through a 50/50 beam splitter and low NA objective lens, respectively, while the reflected light was filtered by a linear polarizer arranged orthogonal to the incident polarization. This ensures a dark background and hence high contrast by blocking unrotated or 180°-rotated fields from areas outside the HCG. To electrically control the colors, we integrated the HCG into an electrochemical cell comprising a Pt working electrode (WE), ITO counter electrode (CE), and electrolyte composed of copper (II) nitrate trihydrate (1 M; Cu(NO_3_)_2_ ∙ 3H_2_O) dissolved in dimethyl sulfoxide (DMSO) (see Methods). Although use of a reference electrode was prohibited by the miniature cell size, the CV characteristics were largely consistent over repeated cycles. Using e-beam lithography, 50 × 50 μm^2^-sized HCGs with periods from 290 to 510 nm were created from an e-beam-evaporated TiO_x_ film atop of the Pt electrode (see Methods). The pixels were designed with the same slit width used in Fig. 1 and height of 100 nm, predicted to provide a large s-pol spectral shift of ~170 nm between bare and Cu-filled HCG responses (see Supplementary Note 3 and Supplementary Fig. 4).

**Fig. 2:**
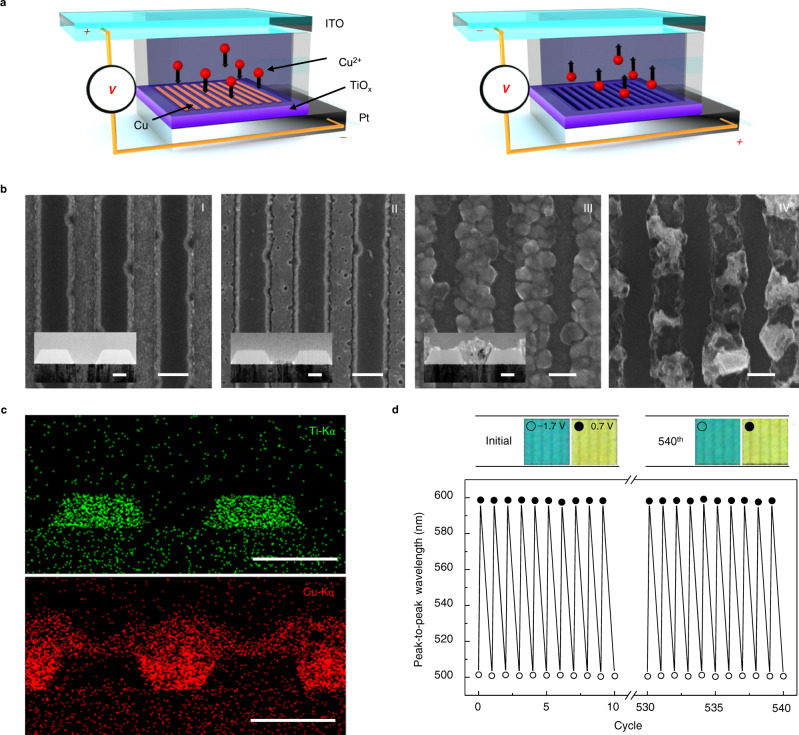
Characterization of electrochemical cell. **a** Schematic of electrochemical cell undergoing Cu electrodeposition from Cu ion reduction (left) and dissolution from Cu oxidation (right) inside the HCG slits in response to an applied potential at the WE. **b** SEM images of HCG during Cu electrodeposition (I–III) and dissolution (IV) stages (scale bar: 200 nm). Inset: TEM images of FIB cross-sectioned HCG (scale bar: 100 nm). **c** Ti-Kα and Cu-Kα EDS images of the HCG at stage III (scale bar: 200 nm). **d** Peak-to-peak wavelengths, from the cross-polarized reflection spectra of an HCG pixel with *p* = 350 nm, versus number of cycles driven with applied potentials alternating between −1.7 and 0.7 V. Optical microscope (OM) images of HCG pixel at the 1st and 540th cycle (top). Bare and partially Cu filled-HCG states are denoted as open and solid circles, respectively.

By applying a voltage across the two electrodes via a potentiostat (Fig. 2a), Cu occupancy in the slits was controlled. Cu deposits inside the slits when a cathodic bias (i.e., potential needed to reduce Cu^2+^ ions to Cu (s)) is applied to the WE. In an open circuit, the Cu dissolves over a few minutes due to the nitric acid in the electrolyte solution^33^. This can be halted with a small negative bias that cancels the excess oxidative current. Dissolution is accelerated with an anodic bias via oxidation. Distinct HCG morphologies throughout these processes are observable from top-view scanning electron microscopy (SEM) images and cross-sectional high-resolution transmission electron microscopy (HR-TEM) (Fig. 2b). On a clean Pt surface within the HCG slits (stage I), Cu accumulates displaying smooth and uniform coverage (stage II). In the event the deposited Cu exceeds the HCG height, it is no longer laterally restricted, and receives an increased ion flux that promotes nucleation of larger (>~100 nm) crystals (stage III). Scanning transmission electron microscopy (STEM) and energy dispersive spectroscopy (EDS) confirm that the HCG and deposited metal are composed of TiO_x_ and Cu, respectively (Fig. 2c). Additional characterization of the Cu is provided in Supplementary Note 4 and Supplementary Fig. 5. Unlike Cu deposition, dissolution occurs spatially sporadically, leaving the metal strip initially disordered (stage IV), the effect of which we describe later.

The use of Pt as the WE electrode, whose chemical stability is well-established, ensures robust and prolonged reversibility over repeated cycles. A step potential alternating between −1.7 and 0.7 V for 3.9 and 24 s, respectively, was repeatedly applied to a 350 nm-period pixel. Peak-to-peak wavelengths in the cross-polarized reflection spectra and associated images of the pixel remain unchanged up to 540 cycles (Fig. 2d), which represent a >2-fold improvement over results from electrodeposited Ag on Au nanodomes, the reversibility of which is limited by the growth of Ag/Au alloys^11,34^.

### Electrochemical control of Cu morphology and corresponding optical response

**Fig. 3:**
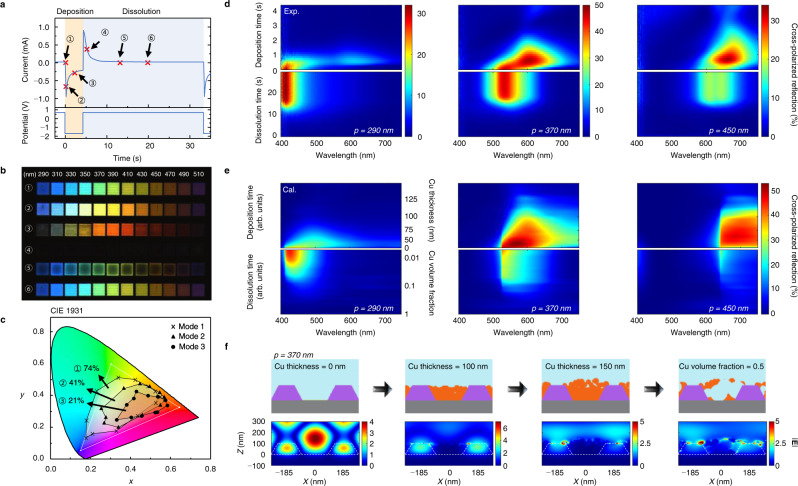
Active color tuning and blackening. **a** Chronoamperometric profile (top) in response to an applied step potential from −1.7 to 0.7 V (bottom). Pixel modes are denoted from 1 to 6 according to the elapsed time: 0, 0.4, 2.0, 5.2, 13.0, 20.0 s. **b** Cross-polarized OM images of pixels with periods from 290 to 510 nm displaying the six different pixel modes. **c** CIE 1931 chromaticity of pixel mode 1 (cross), 2 (solid triangle), and 3 (solid circle), each labelled by their CIE colorspace coverage ratio with respect to the sRGB triangle. **d** Measured cross-polarized reflection spectra during (top) Cu deposition and (bottom) dissolution as a function of time. **e** Simulated cross-polarized reflection spectra for Cu deposition (top) and dissolution (bottom) over time. **d**, **e** From left to right, the pixel period corresponds to 290, 370, and 450 nm. **f** Schematic (top) and corresponding s-pol electric field distribution (bottom) of the HCG featuring an empty slit, Cu-filled slit, slit overfilling with Cu and slit with dissolving Cu, from left to right. The fields were calculated at the resonance wavelengths of 532 and 600 nm for the empty and filled slit, respectively, and at arbitrary wavelengths of 650 and 700 nm for the overfilled and dissolving slit, respectively.

The temporal profile of the measured current in response to an applied voltage (Fig. 3a) provides mechanistic insight into the unique set of operational modes offered by the pixel. We label these modes as 1–6 in the current profile and describe their corresponding cross-polarized reflection images across pixels from *p* = 290 to 510 nm (Fig. 3b, Supplementary Fig.  6, and Supplementary Video 1). All pixels start as bare HCGs covering a wide range of colors defined by the period-dependent s-pol resonance (mode 1). Upon application of a cathodic potential, Cu ions near the exposed Pt surface are immediately reduced, creating a negative spike in the current. Obeying 1D diffusion-dominated reduction in response to a step potential^35^, this current then decays as *t*^−0.5^ (Supplementary Fig. 7) from a decreased ion concentration gradient at the deposited surface that lowers the incoming flux of reducible Cu ions. The resultant rising Cu surface redshifts and broadens the response via s- and p-pol modal contributions within the unfilled parts of the HCG as described earlier (mode 2). As the accumulated Cu reaches the HCG height (mode 3), the colors are determined by the s-pol resonance of the Cu-filled HCG, shrinking the CIE color space coverage by ~72% (Fig. 3c). We note that if the Cu thickness exceeds the HCG height, eventually forming a rough connected film, the HCG birefringence is annulled, eliminating the pixel intensity. This, however, is not our preferred ‘off’ mode because once formed, the Cu on the HCG bars cannot be easily removed due to poor charge transfer.

Applying a step potential of 0.7 V at 4.3 s reverses the current, described by a spike in positive current followed by an exponential decay (Fig. 3a). This decay represents the progressive removal of Cu that leaves the Cu increasingly porous, transitioning the pixels to a black mode (mode 4). For highly disordered systems, it has been shown that energy can be equally partitioned over the wavelengths with similar coupling efficiencies^36,37^, rendering the absorption broadband. Optical images of dissolving Cu-filled pixels without the polarizers confirm this effect through a marked darkening. We note, however, that the images do not show a fully black state (Supplementary Fig. 8a) because the disorder occurs only within the HCG slits (i.e., ~45% of the pixel area). The black state is achieved with the aid of crossed polarizers due to polarization scrambling by the porous Cu (Supplementary Fig. 8b) which further reduces the cross-polarized reflection and yields maximum on/off switching contrasts ranging from ~78 to ~97% (Supplementary Fig. 9). As the oxidative current approaches zero (mode 5), a potential difference across the area of each pixel due to the nontrivial resistivity of the porous Cu (Supplementary Note 5, Supplementary Figs. 10 and 11) causes faster removal of Cu near the edges than the center. Eventually, the pixels return to mode 1 (mode 6). The measured cross-polarized reflection spectra of selected pixels with *p* = 290, 370 and 450 nm displayed over deposition and dissolution time (Fig. 3d) provide a clearer description of the spectral changes, whose qualitative trends from mode 1–3 agree well with our earlier analytical calculations. Peak wavelength shifts of 199, 70 and 53 nm were observed for the three pixels with the corresponding chromaticities shown in Supplementary Fig. 12. These shifts are comparable to recent achievements from metal electrodeposition and ion intercalation designs while the measured on/off contrasts are among the highest when compared to those based on electrochromic polymers (Supplementary Note 6 and Supplementary Fig. 13).

Finite-difference time-domain (FDTD) simulations of the cross-polarized 0th order reflection spectra (Fig. 3e) using the models schematically illustrated in Fig. 3f confirm the bottom-up filling and random removal of Cu during the reductive and oxidative steps. A plane wave source and laterally periodic boundary conditions were used to capture all processes while avoiding the large computational demand associated with finite simulation volumes.

During deposition, nanosized Cu grains were assumed to fill the slits by accumulating from the bottom. Excess Cu deposited above the HCG height was modeled to grow in the form of a semi-elliptical cylinder to reflect the cross-sectioned profile in Fig. 2b (III) (See Supplementary Fig. 14). To mimic the disordered morphology of Fig. 2b (IV) during dissolution, randomly distributed ~30–140 nm-sized pores were continuously introduced until the Cu was completely removed. For comparison to the time-resolved spectra of Fig. 3d, the temporal evolution of the simulated spectra during deposition and dissolution is shown in Fig. 3e, described in detail in Supplementary Note 7 and Supplementary Fig. 15. The simulated trends qualitatively agree with the measured results (Fig. 3d), supporting the modeled mechanisms. Discrepancies are also observable, one of which is the sharper 1st order diffraction onset for the simulation. This is because the model uses periodic boundary conditions and a planewave source whereas our measurements probe a finite-sized grating through a low NA objective lens. Also, the signal attenuation behavior is different during excess Cu deposition above the HCG as our model does not capture the full structural details of the overgrown Cu. Finally, the reflection intensities from the measurements are slightly less than the simulated results because the actual pixel size is finite and sources of far-field loss in the electrochemical cell are not considered in the simulation.

Broadband absorption that attenuates the intensity is verified (Fig. 3f) in the field maps of the upper parts of the overgrown Cu and porous Cu, displaying locally concentrated fields at arbitrary wavelengths. These indicate sites of optical loss arising from the rough morphology. In practice, overfilling the HCG slits with Cu is impractical because it slows the dissolution kinetics and attenuates the tuned colors (See point 4 in Supplementary Fig. 6). On the other hand, slightly underfilling the slits with Cu expedites the dissolution and keeps the tuned colors bright. In fact, for deposited Cu thicknesses much less than the HCG height, dissolution results in direct color restoration bypassing the black color, as the limited porous Cu volume provides fewer sites for plasmonic absorption (Supplementary Fig. 16).

Unlike the results of Fig. 1f, the simulated spectral shifts from the *p* = 370 and 450 nm pixels are less than ~100 nm. We also see that the *p* = 290 nm pixel turns black before the Cu thickness reaches the HCG height. These differences are attributed to the trapezoidal shape of the actual HCG bars compared to the rectangular shape used in Fig. 1, which effectively constricts the resonance field maxima inside the HCG bars and enlarges them inside the slits (Fig. 3f). This is equivalent to increasing the slit width of the rectangular HCG, which reduces the spectral shift between bare and Cu-filled states (Supplementary Fig. 17). At smaller periods (e.g., *p* = 290 nm), the constriction grows to the extent that *β*_0_ is suppressed even before the Cu reaches the HCG height.

### Demonstration as display elements

**Fig. 4:**
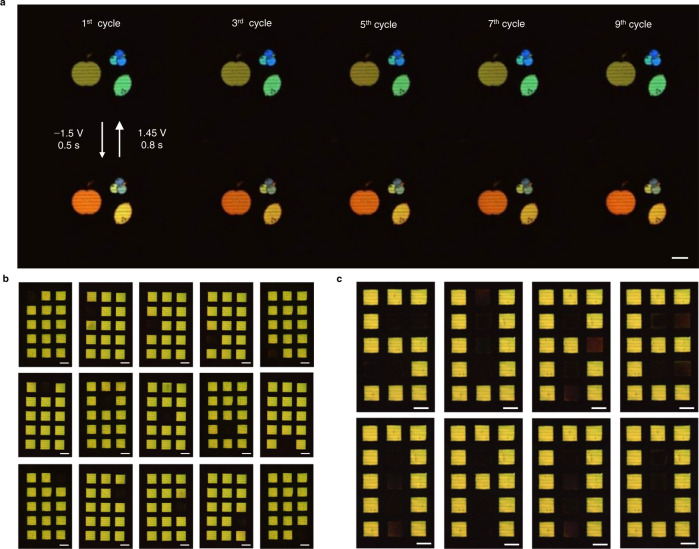
Demonstration of active color display. **a** Cross-polarized OM images of an apple, blueberries, and lemon, switching color in response to an applied potential alternating between −1.5 V and 1.45 V for 0.5 and 0.8 s, respectively, acquired at odd cycle numbers. **b**, **c** Cross-polarized OM images of a 3 × 5 pixel matrix showing (**b**) each pixel sequentially turned off and on and (**c**) letters by turning selected pixels off, proving its feasibility as an alphabetic display (all scale bars: 50 μm).

To highlight the practical applicability of our pixels, we present two types of demonstrations. The first describes a color-switching display that exploits the active tunability of the pixel colors and the second showcases a 3 × 5 pixel matrix featuring on/off switching. We fabricated fruit-shaped pixels with sizes between 30 and 90 μm, displaying green and blue colors passively encoded using HCGs with *p* = 290–450 nm (Fig. 4a). By applying a step voltage of −1.5 V for 0.5 s, the fruits change colors to orange and yellow, and rapidly return to their original state by reversing the voltage to 1.45 V for 0.8 s. This sub-second color tuning can be repeated with excellent regularity (see Supplementary Video 2) and without switching off due to the limited porous Cu volume. To switch off the tuned color, a larger potential of −1.9 V was applied for 3 s to increase the initial Cu volume, followed by an oxidative potential (See Supplementary Fig. 18 and Supplementary Video 3).

For our second demonstration, an individually addressable 3 × 5 pixel matrix comprising 50 × 50 μm^2^ sized HCGs with *p* = 400 nm was prepared. Details of the fabrication procedure and the multichannel current control are provided in Supplementary Note 8, Supplementary Figs. 19 and 20. To demonstrate on/off switching of the untuned color from each pixel, a short step potential of −1.7 V was applied for 0.3 s to quickly predeposit Cu within the HCG slits, followed by a reverse potential of 0.7 V for 2.2 s to dissolve it and yield the porous morphology. To sustain the off state, a negative potential of −0.37 V that cancels the excess cell potential driving the acidic dissolution of Cu must be applied (Supplementary Fig. 21). Fig. 4b and Supplementary Video 4 demonstrate selected pixels of the matrix sequentially turn off. By biasing multiple pixels, the same matrix displays the words ‘SURE’ and ‘NANO’ (Fig. 4c and Supplementary Videos 5–6), proving its feasibility as an alphabetic display. We note that the current performance is limited by cross-talk driven by potential gradients across pixels, which may be addressed by adding diffusion barriers that act as isolation banks. Our designs achieve switching rates up to 2.17 and 0.48 s^−1^ for the on and off state, respectively, and color tuning rates up to 2 and 1.25 s^−1^ for the change and return, respectively. These speeds are comparable or even faster than those of previous metal electrodeposition designs^1^, and can be further enhanced by increasing the electrolyte conductivity or decreasing the pixel area to improve the switching kinetics. We also note that the current design relies on the use of separate polarizers for incident and reflected light. Although this is simple to implement in a microscope, it is not so straightforward in an actual display where space is major constraint. To this end, ‘Janus’ metasurfaces are a promising solution, where different optical functions are encoded for light of opposite propagation directions via meta-atoms with broken out-of-plane symmetry in a thin sheet^38^. Such a metasurface supporting asymmetric transmission (e.g., forward-propagating linearly polarized light and backward-propagating orthogonally polarized light)^39^ offers a possible alternative to our cross-polarized scheme.

In summary, we have demonstrated a dynamic structural color pixel that achieves actively tunable colors and on/off switching through distinct optical mechanisms entailing the electrochemical redox of Cu ions on the exposed parts of a Pt electrode below an HCG. Modal interference of waveguide-array modes in the HCG and the status of the second s-pol mode which can be ‘muted’ or ‘unmuted’ by the Cu occupancy determine the bare and Cu-filled HCG colors under our cross-polarized imaging setup. This mechanism contrasts with those based on surface plasmon polaritons and yields vibrant colors free of plasmonic loss. Conversely, the initial disorder accompanying Cu dissolution yields an off state via broadband plasmonic absorption and depolarized scattering. In addition to the superior chemical stability of Pt, high contrast, and low operational potential (ΔV < 3 V), our pixels offer the key functions needed to promote actively tunable structural colors as dynamic display elements.

## Methods

### Pixel fabrication

A sub-1nm thick Ti wetting layer and ~170 nm-thick Pt layer were sequentially DC sputtered onto a wet oxidized Si wafer (8000 Å) in an Ar atmosphere. Negative (ma-N 2403) or positive resist (PMMA A4) with thicknesses between 300 and 350 nm were spin-coated on top of the Pt substrate. Using e-beam lithography, repeating rows of 10 μm-long grating patterns were written to create 50 × 50 μm^2^-sized pixels. Depending on type of resist, substrates were either developed with AZ300MIF for 60 s or a 3:1 mixture of IPA and water for 45 s. 100 nm-thick TiO_x_ was e-beam evaporated onto the developed substrates at a 0.04 nm/s deposition rate, followed by lift-off.

### Electrochemical cell fabrication

A Pt WE supporting the pixels and ITO CE with two drilled holes (0.75-mm-diameter) for electrolyte injection were separated by a 60-μm-thick thermal film (Meltonix 1170-60) whose central volume was cut out to host the electrolyte. Adhesion between the components was ensured by heating the film up to 135 °C. Electrolyte composed of copper (II) nitrate trihydrate (1 M; Cu(NO_3_)_2_ ∙ 3H_2_O) dissolved in dimethyl sulfoxide (DMSO) was injected into the cell via one of the holes in the CE using a micropipette. Air was simultaneously drawn from the other hole with a separate micropipette to avoid trapped bubbles in the cell. Adhesive Cu tape was attached to each end of the WE and CE to provide electrical connection to the potentiostat (CompactStat.h, IVIUM) terminals.

### Optical measurement

Cross-polarized reflection was measured with a home-built confocal microscope. White light from a halogen lamp was delivered through a linear polarizer and normally illuminated onto the pixels inside the electrochemical cell. The reflected signal was filtered by a separate polarizer oriented orthogonal to the original one. To obtain an image, a 0.3 NA objective lens was used to collect and deliver light into a CCD (STC-TC202USB-AS, SENTECH). For the spectrum, a 0.15 NA objective lens and spectrometer (Acton SP2300, Princeton Instruments) fiber-coupled to the pinhole were used.

### Characterization

Top-view spatial analysis of the pixels was performed with a field-emission scanning electron microscope (JSM-6700F, JEOL) at an operating voltage of 15.0 kV. Side-view analysis was performed by cross-sectioning the HCGs via focused ion beam milling (crossbeam 540, ZEISS). Lattice fringes were acquired with a field-emission transmission electron microscope (JEM-2100F, JEOL) while EDS information was acquired in scanning transmission electron microscopy (STEM) mode, at an operating voltage of 200 kV.

### Calculations

FDTD simulations were performed to compute the cross-polarized reflection and electric field distributions, while rigorous coupled-wave analysis (RCWA) was used to obtain the 0th order s and p-pol reflection efficiencies. For the FDTD simulations, a planewave light source was normally injected onto a grating rotated 45° with respect to the incident electric field vector. Periodic and perfectly matched layer boundary conditions were used for the x–y and z dimensions, respectively. Rectangular side-profiles were used to model the grating and fully deposited Cu for the modal analysis of Fig. 1 while trapezoidal gratings and granular Cu nanoparticles were used to simulate the experimental conditions of Fig. 3. To obtain the s and p-pol electric field distributions, planewave sources with light perpendicular and parallel to the grating vector were injected. A mesh volume of 2 × 2 × 2 nm^3^ was used to model the grating and Cu.

For the bare HCG analysis, a modal expansion method was used, with details provided in Supplementary Note 1. The modal profile in Fig. 1b was calculated at a 550 nm wavelength. Surface impedance boundary conditions (SIBC) were used for analyzing the Cu-filled HCG.

## Supplementary information

Supplementary Info

Description of Additional Supplementary Files

Supplementary Movie 1

Supplementary Movie 2

Supplementary Movie 3

Supplementary Movie 4

Supplementary Movie 5

Supplementary Movie 6

## Data Availability

Data presented in this publication is available on Figshare with the following identifier (10.6084/m9.figshare.19771957.v1).
