# Supplementary material for: Active electrochemical high-contrast gratings as on/off switchable and color tunable pixels
Source: Nat Commun. 2022 Jun 13;13:3391. doi: 10.1038/s41467-022-31083-z (PMC9192692; doi:10.1038/s41467-022-31083-z)
Supplement: Supplementary file 1 — Supplementary Info [file 41467_2022_31083_MOESM1_ESM.pdf]

Supplementary Information

Active electrochemical high-contrast gratings as on/off switchable and color tunable pixels

Cheon Woo Moon[1, 2]*, Youngji Kim*[1, 2] *and Jerome Kartham Hyun*[1]*

[1] Department of Chemistry and Nanoscience, Ewha Womans University, Seoul 03760, Republic of Korea
[2] These authors contributed equally

E-mail: kadam.hyun@ewha.ac.kr (Jerome Kartham Hyun)

Table of Contents

Supplementary Notes 1-8

Supplementary Figures 1-21

Supplementary References

Supplementary Note 1. Analytical calculation of waveguide-array modes in bare and Cu-filled HCGs

To calculate the dispersion and modal profile of the waveguide-array modes in a bare HCG, we used the modal formalism developed by the Chang-Hasnain group.[1] We note that the dispersion and modal profile only depend on the lateral dimension (defined as the x direction) and optical constants of the HCG bar and slit, in contrast to the reflection, which additionally depends on the HCG height and material above and below the HCG. Here, our treatment focuses on s-pol light under normal incidence, which restricts our solution to even modes.

In Fig. 1b, the grating bar width (s), slit width (a), and period (p) are 205 nm, 165 nm, and 370 nm, respectively. The lateral magnetic and electric fields ($\mathcal{H}_{\mathrm{x,m}}(x)$ and $\mathcal{E}_{\mathrm{y,m}}(x)$) inside the HCG are expressed as follows.

$$\mathcal{H}_{\mathrm{x,m}}(x) = \cos\left(\frac{k_{\mathrm{s,m}}s}{2}\right)\cos\left(k_{\mathrm{a,m}}\left(x-\frac{a}{2}\right)\right),\ \ 0<x<a \qquad (1\text{-}1)$$

$$\mathcal{H}_{\mathrm{x,m}}(x) = \cos\left(\frac{k_{\mathrm{a,m}}a}{2}\right)\cos\left(k_{\mathrm{s,m}}\left(x-\frac{a+p}{2}\right)\right),\ \ a<x<p \qquad (1\text{-}2)$$

$$\mathcal{E}_{\mathrm{y,m}}(x) = -\frac{k_0}{\beta_{\mathrm{m}}}\sqrt{\frac{\mu_0}{\varepsilon_0}}\mathcal{H}_{\mathrm{x,m}}(\mathrm{x}),\quad 0<x<p \qquad (1\text{-}3)$$

where μ_0, and ε_0 are the vacuum permeability, and vacuum permittivity, respectively. k_{s}, k_{a}, and k_0 are the lateral wavenumbers inside the grating bars, slits, and vacuum, respectively. β_{m} is the propagating constant in the longitudinal direction (z). The modal profile in the HCG can be found from equations (1).

By matching the boundary conditions, the characteristic equation is found as:

$$k_{\mathrm{s,m}}\tan\left(k_{\mathrm{s,m}}\frac{s}{2}\right) = -\,k_{\mathrm{a,m}}\tan\left(k_{\mathrm{a,m}}\frac{a}{2}\right). \qquad (2)$$

We can then find β_{m} from

$$\beta_{\mathrm{m}}^2 = (\frac{2\pi n_{\mathrm{slit}}}{\lambda})^2 - k_{\mathrm{a,m}}^2 = (\frac{2\pi n_{\mathrm{bar}}}{\lambda})^2 - k_{\mathrm{s,m}}^2, \quad (3)$$

where n_{slit} and n_{bar} are the refractive indices of slit and grating bar, respectively.

To calculate the waveguide-array modes in a Cu-filled HCG, we implemented surface impedance boundary conditions (SIBC) first proposed by Lochbihler et al.[2,3] The SIBC treatment was previously used to understand the effect of extraordinary optical transmission through sub-wavelength slits in metals.[3-6] Similarly, here, the modes in a Cu-filled HCG can be easily found by ignoring the field inside the Cu and assuming that the tangential components of the electric and magnetic fields are related as follows.

$$\boldsymbol{E}_{\parallel} = \varepsilon_{\mathrm{Cu}}^{-1/2}\, \boldsymbol{n} \times \boldsymbol{H}_{\parallel} \quad (4)$$

, where $\varepsilon_{\mathrm{Cu}}$, and $\boldsymbol{n}$ are the Cu permittivity and normal unit vector, respectively. Here, the s-pol lateral electric field can be expressed as follows.

$$\mathcal{E}_{\mathrm{y,m}}(x) = \eta\cos(k_{\mathrm{s,m}}(x-a)) + k_{\mathrm{s,m}}^{-1}\sin(k_{\mathrm{s,m}}(x-a)), \quad a < x < p \quad (6)$$

$$\eta = \frac{i}{k_0\sqrt{\varepsilon_{\mathrm{Cu}}}}. \quad (7)$$

We can find $k_{\mathrm{s,m}}$ as follows (8):

$$\tan(k_{\mathrm{s,m}}s) = \frac{2\eta k_{\mathrm{s,m}}}{(k_{\mathrm{s,m}}\eta)^2 - 1} \quad (8)$$

Using these set of equations, we calculated the modal profiles of the bare and Cu-filled HCG at λ=550 nm.

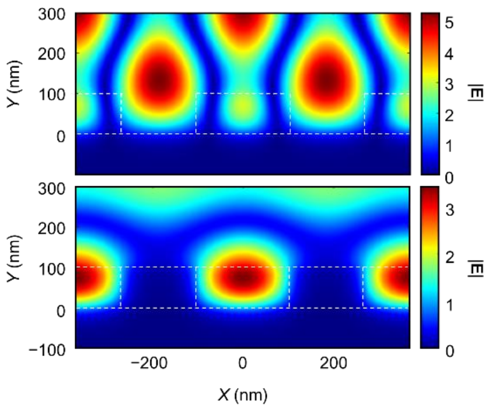

Supplementary Figure 1 | Electric field distributions of bare and Cu-filled HCG. Cross-sectional view of s-pol electric field distributions of a bare (top) and Cu-filled HCG (bottom) at its respective resonance wavelength of 526 and 684 nm. The HCG height, period, and slit width are 100, 370, and 165 nm, respectively.

Supplementary Note 2. Analytical study of partially Cu-filled HCGs

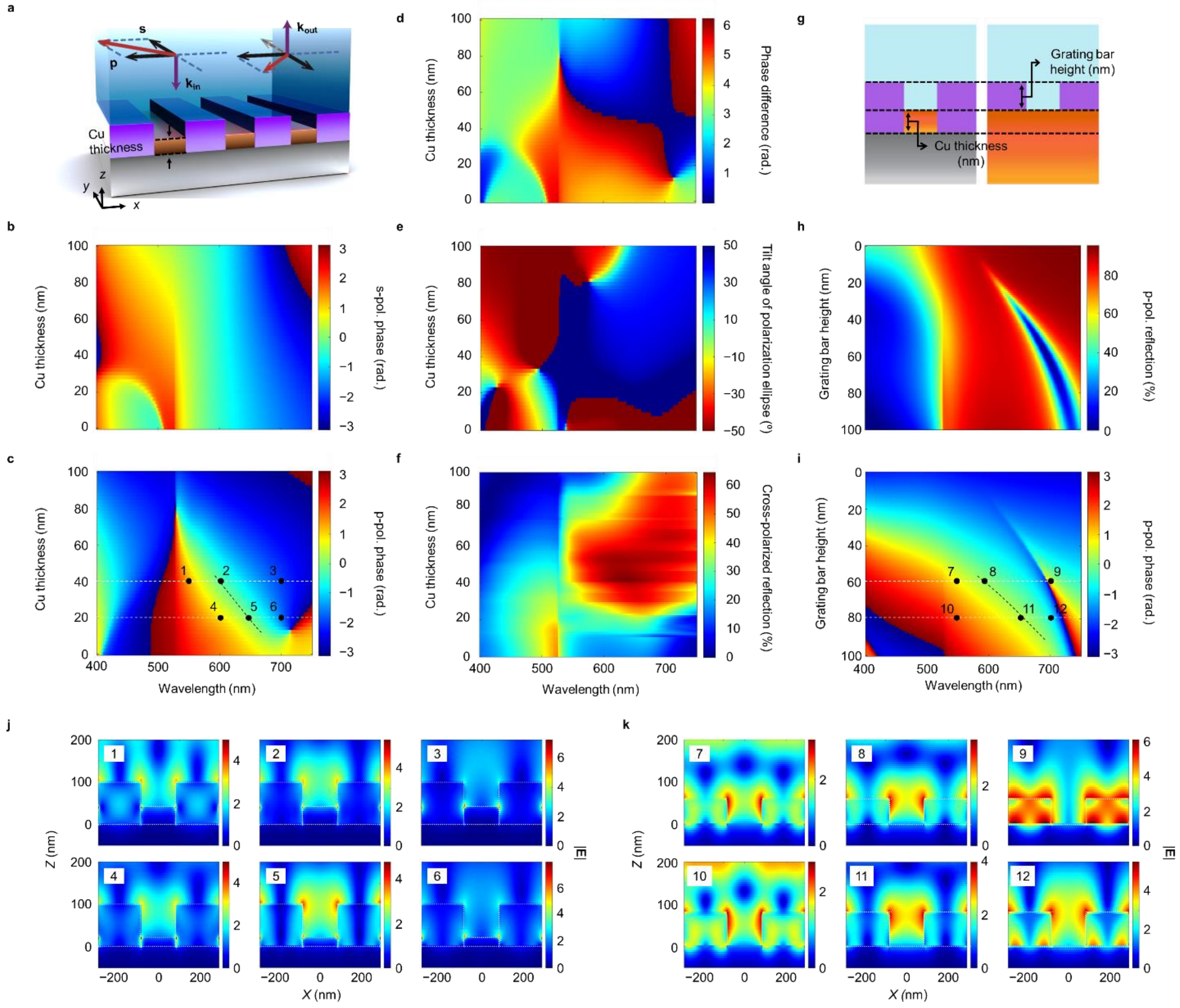

Supplementary Figure 2 | Analytical calculations of partially Cu-filled HCGs. a, Schematic of partially Cu-filled HCGs. Cu is partially filled in the slits between the HCG bars. **b,c,** Phase under s-pol (**b**) and p-pol (**c**) light as a function of Cu thickness and wavelength. Points 1-3 indicate a Cu thickness of 40 nm and wavelength of 550, 600, and 700 nm. Points 4-6 indicate a Cu thickness of 20 nm and wavelength of 600, 650, and 700 nm. **d-f,** Phase difference between s-pol and p-pol responses (**d**), tilt angle of polarization ellipse (**e**), and cross-polarized reflection (**f**) as a function of Cu thickness and wavelength.

g, Schematic of bare HCG on Cu substrate which mimics the partially Cu-filled HCGs. When the Cu thickness of a partially Cu-filled HCG increases (left), a similar p-pol response is observable for a bare HCG on a Cu substrate with decreasing HCG bar height (right). **h,i,** Reflection (**h**) and phase (**i**) spectra under p-pol light as a function of grating bar height for a bare HCG on Cu substrate. Points 7-9 indicate a HCG bar height of 60 nm and wavelength of 550, 590, and 700 nm. Points 10-12 indicate a HCG bar height of 80 nm and wavelength of 550, 650, and 700 nm. **j,k,** Electric field distribution under p-pol light for the (**j**) partially Cu-filled HCG at selected points in **c**, and (**k**) bare HCG on Cu substrate at selected points in **i**.

Unlike the bare and Cu-filled HCGs where the s-pol response dominates the optical properties, the partially Cu-filled HCG (**a**) is affected by both s-pol and p-pol responses. The fundamental p-pol waveguide-array mode, propagating within the bare parts of the HCG, carries field concentrated at the slit sidewalls, and is therefore reflected by Cu accumulated inside the slit. This means that for thicker Cu, the mode wavelength must be shorter to accumulate the same phase over the propagation length. The calculated p-pol 0th order reflection phase spectra plotted over Cu thickness up to the grating height confirm that the characteristic phase at zero radians blueshifts with increasing Cu thickness (**c**). On the other hand, the s-pol phase at 0 radians is relatively unaffected by the Cu thickness in the adjacent slits (**b**) due to its field concentrated inside the HCG bar (Fig. 1b). The two distinct s and p-pol phase dependences on the Cu thickness, respectively, result in a ~π phase difference and 45° elliptical polarization tilt angle over a range of wavelengths and Cu thicknesses (**d, e**). This translates to a cross-polarized reflection peak (**f**) much broader

than that of the bare or Cu-filled HCG because both s and p-pol responses contribute to the reflected field vector rotation, rather than just the s-pol response. These properties are also confirmed with a bare HCG on Cu substrate whose p-pol response mimics the partially Cu-filled HCG (**g**). Here, an increasing Cu thickness for a partially Cu-filled HCG gives a p-pol response similar to a bare HCG on Cu substrate with decreasing HCG bar height since both scenarios describe a decreasing propagation length for the p-pol mode in the bare part of the HCG. Indeed, for the bare HCG on Cu substrate, the phase transition across 0 radians blueshifts with decreasing HCG bar height (**i**). Electric field distributions under p-pol illumination also confirm similar interference fields concentrated at the bare part of the slit sidewalls at the phase transition, for both partially Cu-filled (**j**) and bare HCG on Cu substrate (**k**). This field pattern is clearly distinct from those at non-resonant conditions (1,3,4, and 6) shown in (**j**) and the surface plasmonic resonances (9 and 12) shown in (**k**).

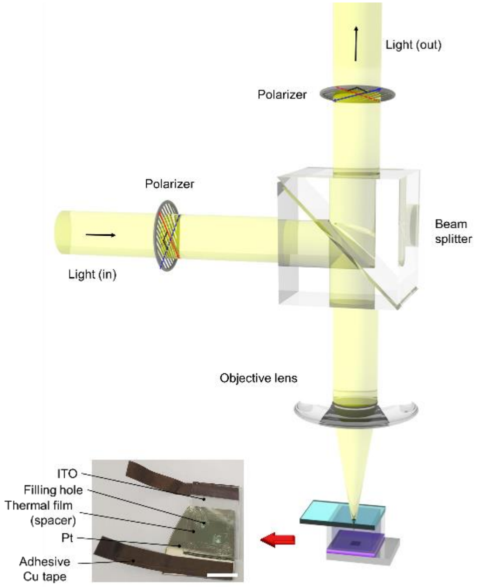

Supplementary Figure 3 | Optical microscope setup and electrochemical cell. Schematic of the optical measurement setup with orthogonally oriented linear polarizers in the incident and reflected beam path. Photo shows the fabricated electrochemical cell (scale bar: 1 cm).

Supplementary Note 3. Optimization of HCG bar height

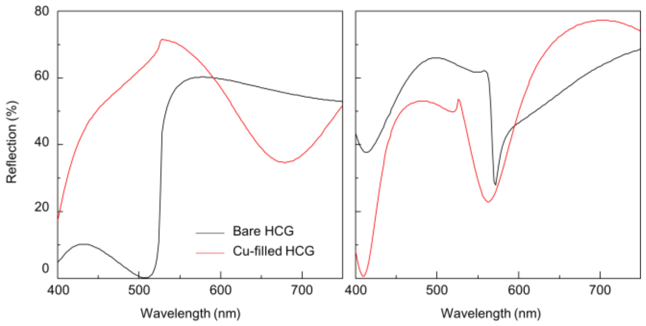

Supplementary Figure 4 | HCG bar height optimization. S-pol 0th order reflection spectra of bare and Cu-filled HCG with HCG bar height and Cu thickness of 100 (left) and 200 nm (right), respectively.

As the grating bar height controls the phase accumulation of the modes, it is an important parameter for optimizing the spectral shift between bare and Cu-filled HCG responses. Calculations reveal that a large s-pol spectral transition of ~170 nm is possible when the grating bar height is 100 nm. In contrast, a non-optimum of height of 200 nm shows little spectral shift, underscoring the importance of grating parameter optimization. Additionally, as shown in Fig. 1c, the bare HCG may display more than one peak and trough for a range of HCG bar heights because of the complexity associated with the interference between dual waveguide-array modes. This compromises the color purity of the passively-encoded color, necessitating careful consideration when choosing the HCG bar height.

Supplementary Note 4. Characterization of electrodeposited Cu in the HCG slits

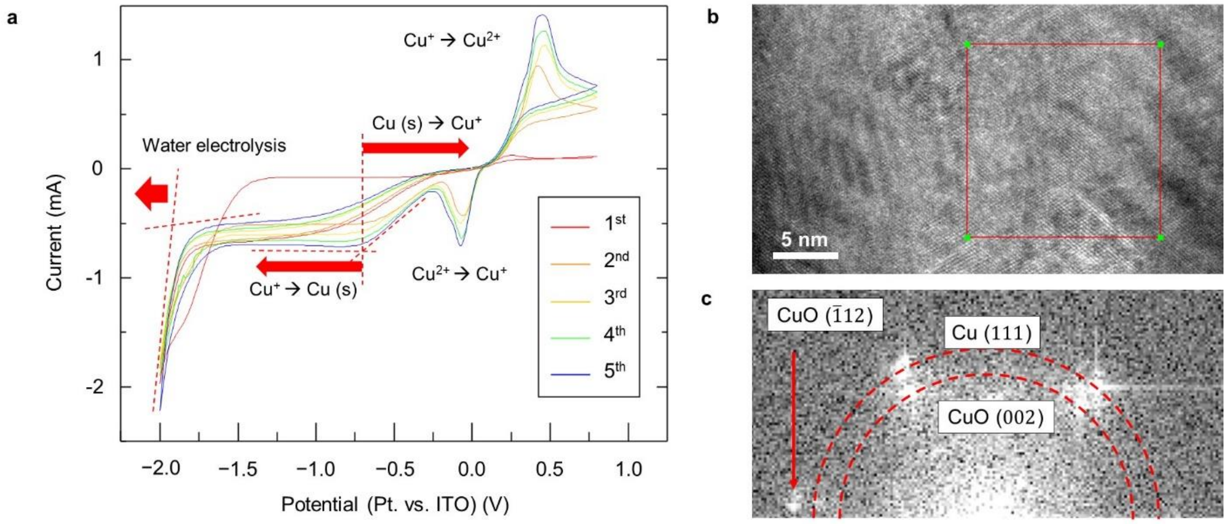

Supplementary Figure 5 | Characterization of electrodeposited Cu in HCG slits. a, Cyclic voltammetry of 1 M concentration $Cu(NO_3)_2 \cdot 3H_2O$ in DMSO. **b,** HR-TEM image of electrodeposited Cu in HCG slit. **c.** FFT pattern of selected area in (**b**).

Cyclic voltammetry was conducted with 1 M $Cu(NO_3)_2 \cdot 3H_2O$ in DMSO between a Pt WE and ITO CE using a 5 mV/s scan rate (**a**). Cyclic voltammetry was performed by sweeping the applied potential from 0 V (Pt vs. ITO) to 0.8 V (Pt vs. ITO) then to –2.0 V (Pt vs. ITO) and back to 0 V (Pt vs. ITO). Note, the potential values assigned to the redox processes from our two-electrode configuration can differ from those measured using a three-electrode configuration (i.e., including a reference electrode) because the interfacial potential driving the redox reactions is not explicitly controlled.[7] Applying a negative voltage larger than −1.9 V (Pt vs. ITO) results in a steep increase of current due to hydrogen gas generation by water electrolysis and is therefore avoided in our experiment. Anodic and cathodic peaks (~−0.1 and ~0.4 V (Pt vs. ITO), respectively) represent conversion of ionic species between Cu^{2+} and Cu^{+} ions ($Cu^{2+} + e^{-} \rightleftharpoons Cu^{+}$). The cathodic region marked by the plateau in current (<−0.7 V (Pt vs. ITO))

corresponds to the reduction of Cu^+ ions ($Cu^+ + e^- \rightarrow Cu$ (s)) while the anodic region (> −0.7 V (Pt vs. ITO)) corresponds to dissolution of Cu (s) into its ions. The current plateau in the cathodic region is presumably due to the limited diffusion of Cu ions from decreased ion diffusivity in DMSO ($D_{DMSO} = 6.7 \times 10^{-6}$ cm^2/s) compared to that in water ($D_{water} = 1.266 \times 10^{-5}$ cm^2/s),[8,9] and the large difference in active area between the working and counter electrode.[10]

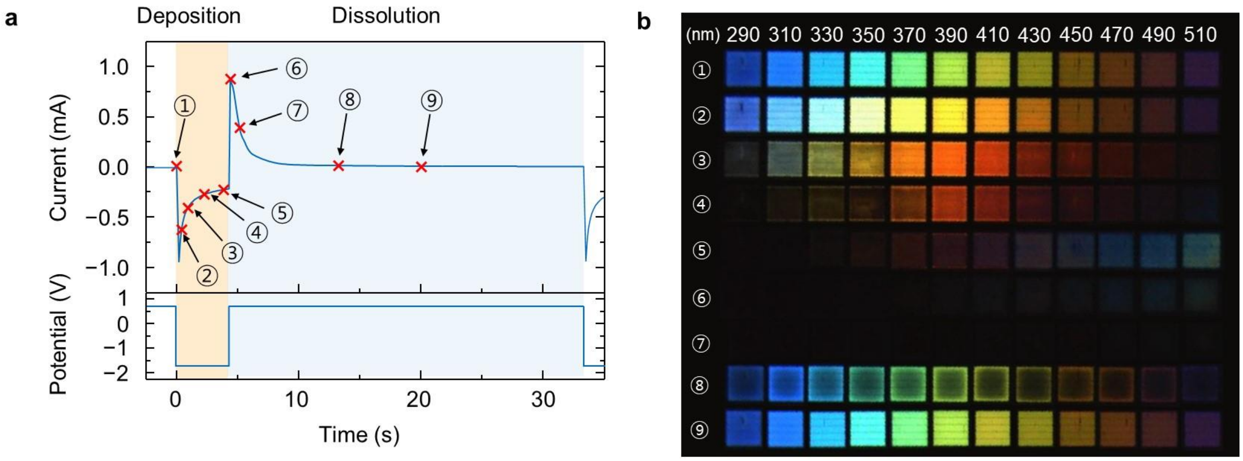

Supplementary Figure 6 | Detailed electrochemical coloration profile of HCG in response to an applied step potential. a, Chronoamperometric profile (top) in response to an applied step potential from −1.7 to 0.7 V (bottom). To describe the color evolution in detail, more points are sampled over time than in Fig. 3a, denoted from 1 to 9. The elapsed times are 0, 0.4, 1.0, 2.0, 4.0, 4.3, 5.2, 13.0, 20.0 s. **b**, Cross-polarized OM images of pixels with periods from 290 to 510 nm at the sampled times.

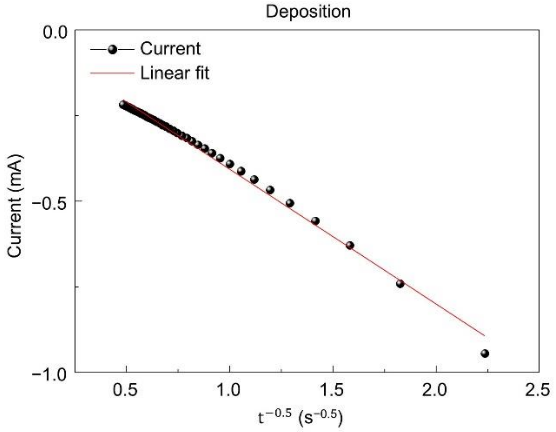

Supplementary Figure 7 | Cottrell plot of the HCG. Current as a function of decay times during electrodeposition fitted with a $t^{-0.5}$ dependence.

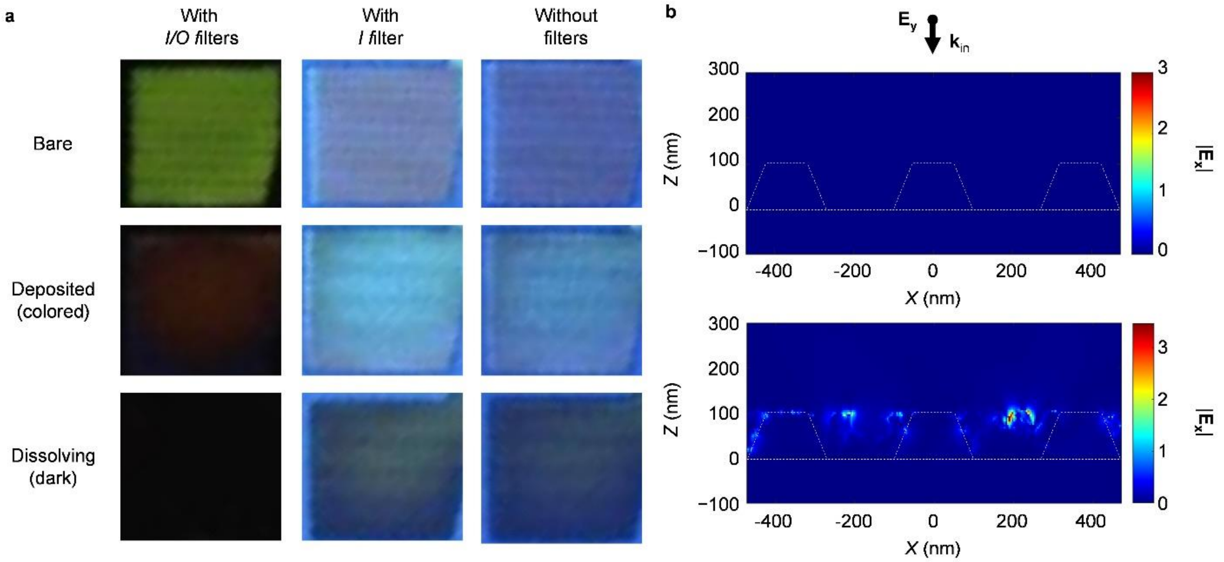

Supplementary Figure 8 | Polarization scrambling. a, Optical microscope images of a 50×50 μm^2 -sized bare, Cu-deposited, and Cu-dissolving pixel with p=400 nm viewed with crossed input (*I*) and output (*O*) polarizers, without *O*, and without *I* and *O*. **b,** Cross-sectional view of simulated |$\mathbf{E_x}$| field excited by s-pol light ($\mathbf{E_y}$) for bare (top) and Cu-dissolving HCG (bottom) at 550 nm. The |$\mathbf{E_x}$| field is not observable for the bare HCG, as expected, but is strong for the Cu-dissolving HCG due to the morphological disorder inherent in the Cu that scrambles the incident polarization.

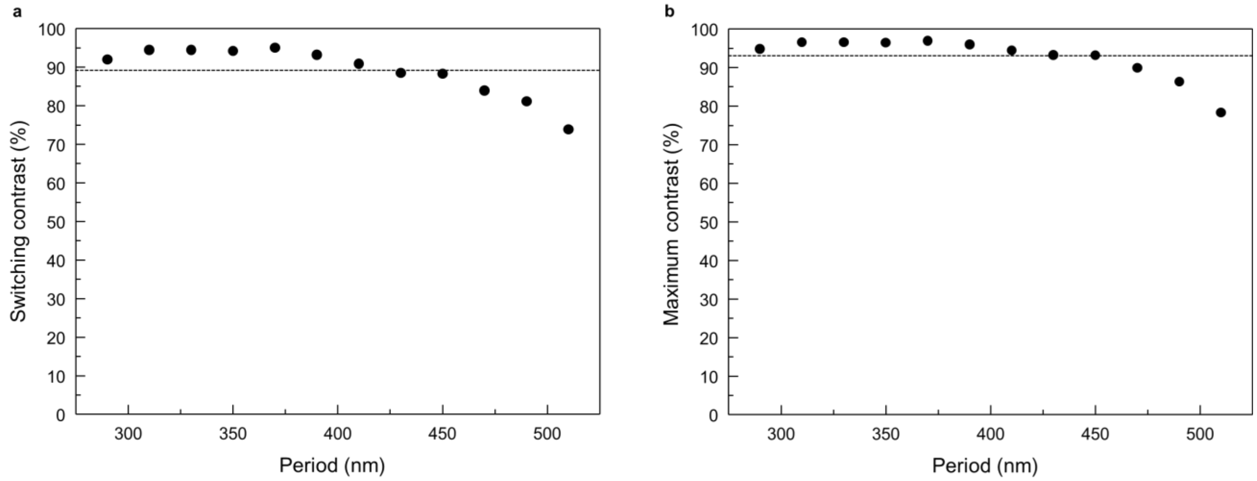

Supplementary Figure 9 | Pixel contrasts displayed in Fig. 3b. a, On/off switching contrasts calculated for all pixels with periods from 290 to 510 nm. **b,** Maximum contrasts calculated for all pixels with periods from 290 to 510 nm. The averaged values from the 12 data points are indicated as dotted lines.

The on/off switching contrast is defined as follows

$$\text{Switching contrast (\%)} = \frac{\int_{400\ nm}^{750\ nm} R(\text{mode1}) - R(\text{mode4})\ d\lambda}{\int_{400\ nm}^{750\ nm} R(\text{mode1})\ d\lambda} \times 100 \tag{14}$$

, where R, t, and λ indicate reflection spectrum, time, and wavelength, respectively.

The maximum contrast is defined as the contrast between the state of maximum brightness and the off state as follows.

$$\text{Maximum contrast (\%)} = \frac{\int_{400\ nm}^{750\ nm} R(\text{max}) - R(\text{mode4})\ d\lambda}{\int_{400\ nm}^{750\ nm} R(\text{max})\ d\lambda} \times 100 \tag{15}$$

Supplementary Note 5. Cu thickness distribution from the drift diffusion equation.

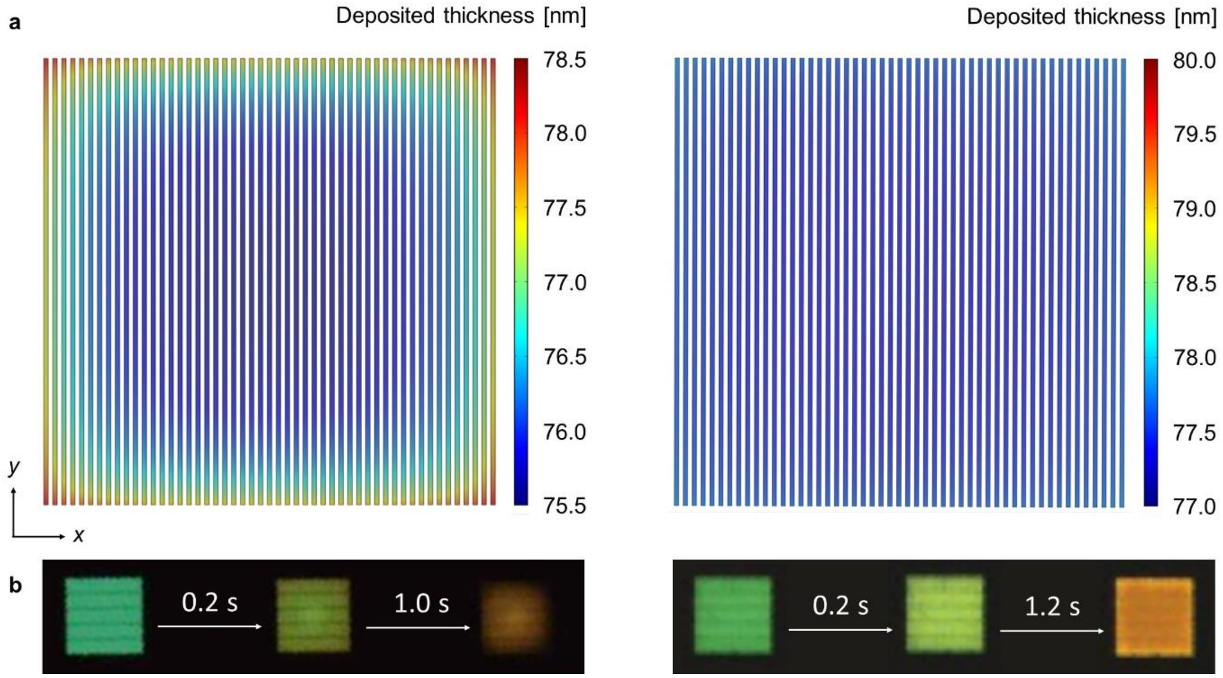

Supplementary Figure 10 | Cu thickness distribution in pixel. a, Simulated thickness distribution of Cu electrodeposited in HCG slits after applying (left) −1.9 V for 5 s and (right) −1.7 V for 30 s. The grating period is 1 μm. **b**, (Left) Sequential images of a 370 nm-period pixel with Cu electrodeposited in HCG slits from an applied voltage of (left) −1.9 V at 0, 0.2 and 1.0 s and (right) −1.7 V at 0, 0.2 and 1.2 s.

For large voltages applied for short durations, the brightness within a single pixel appears non-uniform. To explain this effect, the Cu thickness distribution was simulated by solving the Nernst-Planck equation for a single pixel using the finite element methods software, COMSOL. The Nernst-Planck equation describes the drift-diffusion kinetics of the Cu ions under a static electric field. The simulated pixel size was 50 × 50 μm^2. The grating spacing was 500 nm and the period was 1 μm. A 1 M Cu^{2+} concentration with 50 % Cu^{+} ion conversion efficiency was used. The diffusivity of the electrolyte was 10^{-10} m^2/s. Equilibrium potentials were estimated from the measured CV curve. As shown in Fig. (a, left), the simulated results for a voltage of −1.9 V applied for 5 s reveal more electrodeposited metal along the edge of the pixel than in the center. Electrodepositing for longer durations (30 s) at a lower potential −1.7 V improves the uniformity of the Cu thickness as shown in Fig. (a, right). The simulated results can be qualitatively verified

with measurements as shown in Fig. (b). At large applied voltages applied for short durations (Fig. (b, left)), one notices darker edges appearing over time, indicative of thicker Cu at the edges. On the other hand, for smaller voltages applied for longer durations (Fig. (b, right)), the colors remain spatially uniform over the pixel area, in agreement with the simulated results. The spatial non-uniformity found during dissolution can be explained in a similar manner, where the pixel edges appear brighter than the center, suggesting faster dissolution at the edges than at the center.

We note that the spatial inhomogeneity of the deposited metal over the pixel area is not unique to our pixel, but a general phenomenon for electrodeposition on a resistive electrode. Previous research (ACS Energy Lett. 3, 2823−2828 (2018)) on dynamic windows using metal electrodeposition on a square ITO electrode derived the potential drop (ΔV) with side length $2L$ as a function of position, where J is current density, T is the thickness, and ρ is the resistivity respectively.

$$\Delta V = V_{edge} - V_{spot} = \frac{J\rho}{T}\sqrt{\left(\frac{L^2-x^2}{2}\right)\left(\frac{L^2-y^2}{2}\right)} \tag{9}$$

This analytical equation can be plotted as follows, which is qualitatively similar to the COMSOL distribution of Fig. (a, left). For electrodeposition, the current density is negative ($J < 0$), meaning $V_{edge} < V_{center}$. This means that more metal is deposited at the edge, giving rise to a darker edge. For dissolution, the current density is positive ($J > 0$), meaning $V_{edge} > V_{center}$. This implies that dissolution occurs faster at the edge which gives the appearance of a brighter edge as observed experimentally. We note that the potential drop decreases for lower J and thicker T, implying that lower voltages and longer durations result in less potential differences over the pixel and thus more uniform coverage, in agreement with the simulation results.

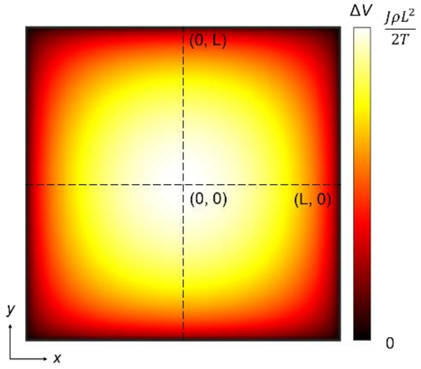

Supplementary Figure 11 | Potential distribution over a $2L \times 2L$ square resistor. Mapping of potential distibution obtained from equation (9).

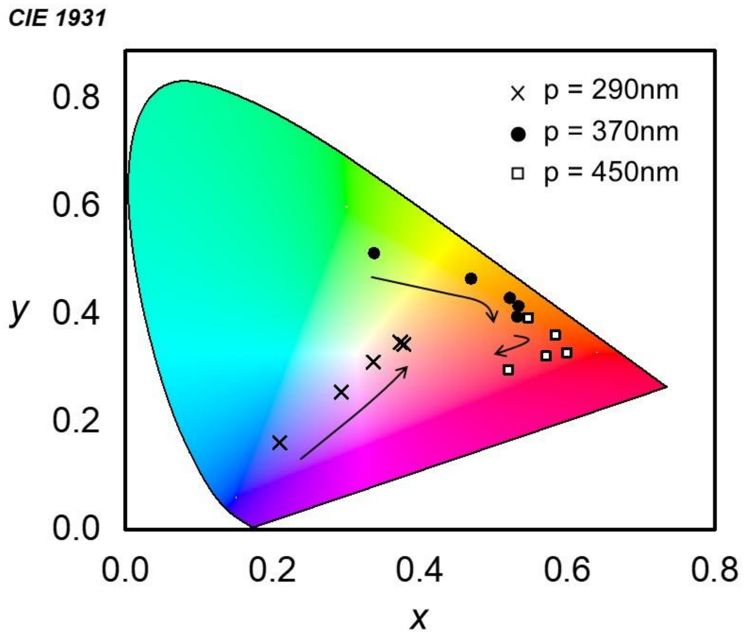

Supplementary Figure 12 | CIE 1931 chromaticities of tuned colors. 290, 370, and 450 nm-period pixel colors generated with −1.7 V applied over 4.2 s.

Supplementary Note 6. Performance comparisons between dynamic reflective structural colors

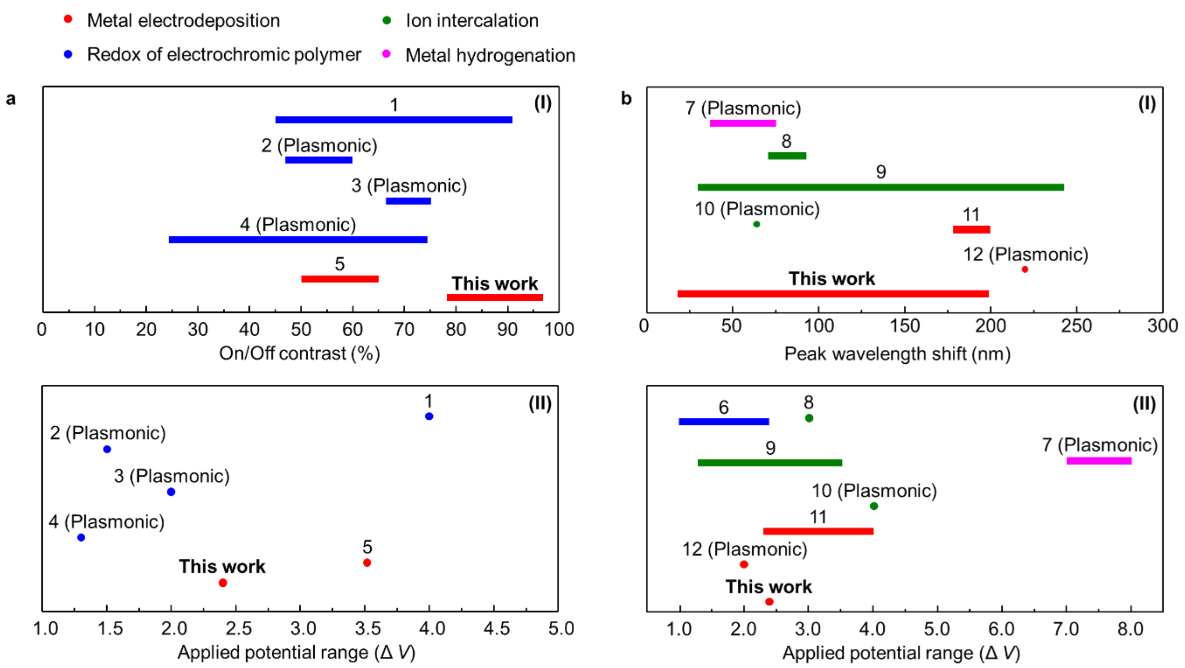

Supplementary Figure 13 | Performance evaluation of reflective dynamic structural colors. a, On/off switching pixels displaying (I) on/off contrast, and (II) applied potential range. **b,** Active color changing pixels displaying (I) peak wavelength shift, and (II) applied potential range. The dynamic coloration mechanisms are metal hydrogenation (pink), redox of electrochromic polymer (blue), ion intercalation (green), and metal electrodeposition (red).

Note 1: The on/off contrast is calculated as,

$$\text{On/off contrast (\%)} = \frac{\int (R_{on} - R_{off})\, d\lambda}{\int R_{on}\, d\lambda} \times 100 \qquad (10)$$

where R_{on} and R_{off} are the reflection spectra for the on and off state, respectively. The wavelength range of interest is the visible range. All values were calculated from data extracted from the reported figures.

Note 2: The peak wavelength shift is calculated as the shift between two given peak wavelengths in the visible range, achievable in a single pixel. For reports providing more than one pixel, a band of wavelengths is provided.

Note 3: The applied voltage is a complex function of extrinsic factors such as the effective electrode size, distance between electrodes, electrolyte concentration, etc, and is therefore only used here to provide a ballpark range rather than a precise measure for comparison.

On/off switching references.

(1) ACS Appl. Nano Mater. 4, 7182–7190 (2021). Electrically Switchable and Flexible Color Displays Based on All-Dielectric Nanogratings, (2) Adv. Mater. 33, 2103217 (2021). Video Speed Switching of Plasmonic Structural Colors with High Contrast and Superior Lifetime, (3) Nano Lett. 17, 7033−7039 (2017). Switchable Plasmonic Metasurfaces with High Chromaticity Containing Only Abundant Metals, (4) Adv. Mater. 28, 9956–9960 (2016). Plasmonic Metasurfaces with Conjugated Polymers for Flexible Electronic Paper in Color, (5) Adv. Mater. Technol. 5, 2000367 (2020). Reflective-Type Transparent/Colored Mirror Switchable Device Using Reversible Electrodeposition with Fabry–Perot Interferometer

Color tuning references.

(6) Adv. Mater. 33, 2101272 (2021). Digital Electrochemistry for On-Chip Heterogeneous Material Integration, (7) Nat. Commun. 10, 5030 (2019). Voltage-gated optics and plasmonics enabled by solid-state proton pumping, (8) Nano Lett. 21, 4343 (2021). Electrochromic Inorganic Nanostructures with High Chromaticity and Superior Brightness, (9) Nat. Commun. 11, 302 (2020). Towards full-colour tunability of inorganic electrochromic devices using ultracompact fabry-perot nanocavities, (10) Nano Lett. 20, 1876 (2020). Plasmochromic Nanocavity Dynamic Light Color Switching, (11) Nat. Nanotechnol. 16, 795–801 (2021). Floating solid-state thin films with dynamic structural colour, (12) ACS Nano 10, 1788−1794 (2016). Mechanical Chameleon through Dynamic Real-Time Plasmonic Tuning

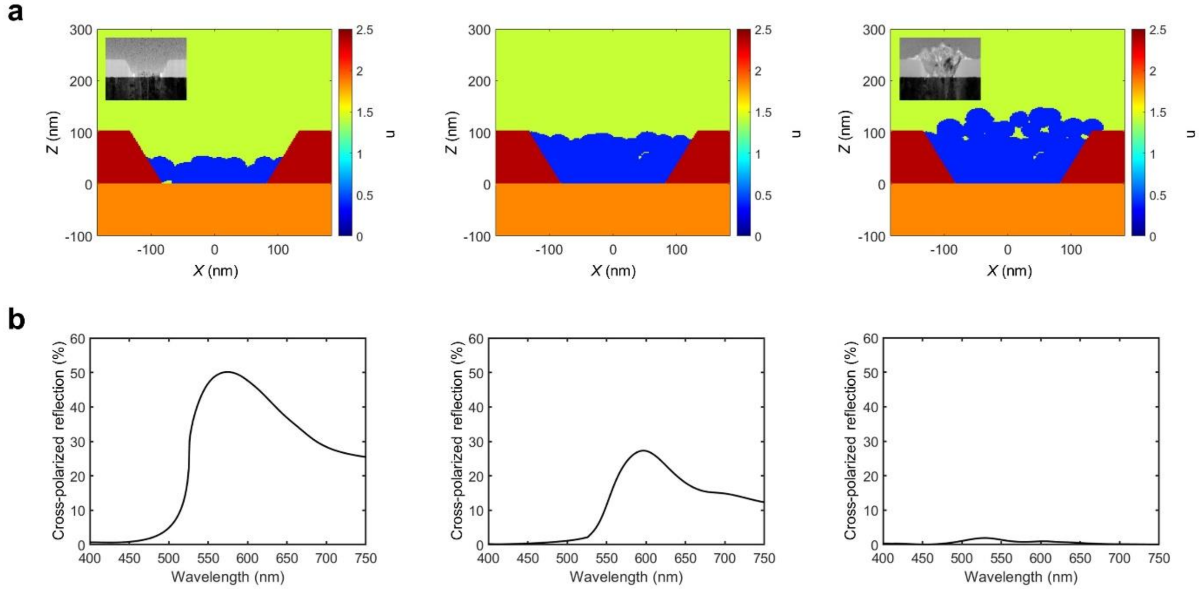

Supplementary Figure 14 | Simulation model for the Cu deposition. a, Spatial map of the real part of the refractive index of the simulated HCG and **b,** simulated cross-polarized 0th order reflection spectra. From left to right, the states correspond to a half-filled (left), fully-filled (middle), and overfilled (right) slit. The two insets in **a** are the corresponding cross-sectioned TEM images.

Supplementary Note 7. Temporal spectral evolution of the simulated deposition process

Cu deposition is modeled as the uniform accumulation of Cu in the HCG slits, whereas Cu dissolution is modeled as the transformation of the deposited Cu into a porous disordered morphology. The simulated reflection spectra for the two scenarios are initially found as a function of Cu thickness and Cu volume fraction, respectively, as shown below.

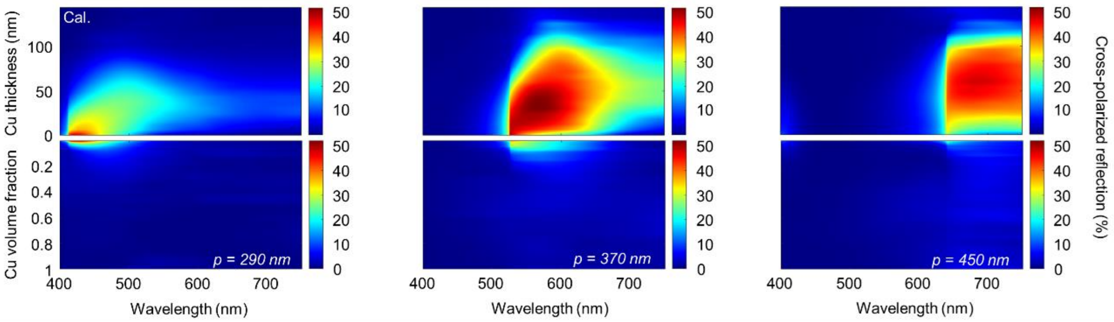

Supplementary Figure 15 | Simulated cross-polarized 0th order reflection spectra over Cu thickness for Cu deposition (top) and Cu volume fraction for dissolution (bottom).

To find the time dependence of the deposited Cu thickness, we first relate the Cu thickness (T_{Cu}) to the current whose time dependence is experimentally measured (See Fig. S3).

$$V = \frac{W}{\rho_m} = \frac{M_w Q}{nF\rho_m} = \frac{M_w}{nF\rho_m}\int I\, dt \tag{11}$$

where W, ρ_m, M_w, Q, n, F, and I are the weight of the deposit, density of metal, atomic weight, total charge, number of electrons, faraday constant, and current, respectively. As a result,

$$\frac{dV}{dt} = \alpha I \tag{12}$$

where α represents the constant $\frac{M_w}{nF\rho_m}$. For the case of electrodeposition, we assume, to a first approximation, that the deposit area, A, does not change with time such that $dV/dt = AdT_{\text{Cu}}/dt$. Therefore,

$$\frac{dT_{\text{Cu}}}{dt} \propto I \propto t^{-0.5} \tag{13}$$

where the $t^{-0.5}$ dependence is found from the Cottrell plot. From this relation, the time dependence of T_{Cu} can be expressed as $T_{\text{Cu}} \propto \sqrt{t}$. For the case of dissolution, the current decays exponentially. Therefore, the Cu volume has an exponential dependence on time.

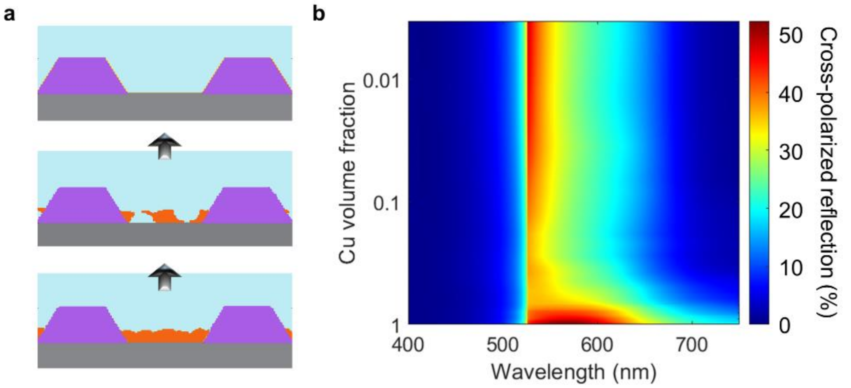

Supplementary Figure 16 | Color tuning with HCG slits underfilled with Cu. a, Schematic of color tuning initiated with underfilled HCG slits with p = 370 nm. **b,** Simulated cross-polarized 0th order reflection spectra for varying Cu filling fraction. Here, a value of 1 corresponds to an initial Cu thickness of 35 nm. Due to the limited porous Cu volume, absorption is not strong and therefore the pixel does not undergo a black state (i.e., it does not turn off).

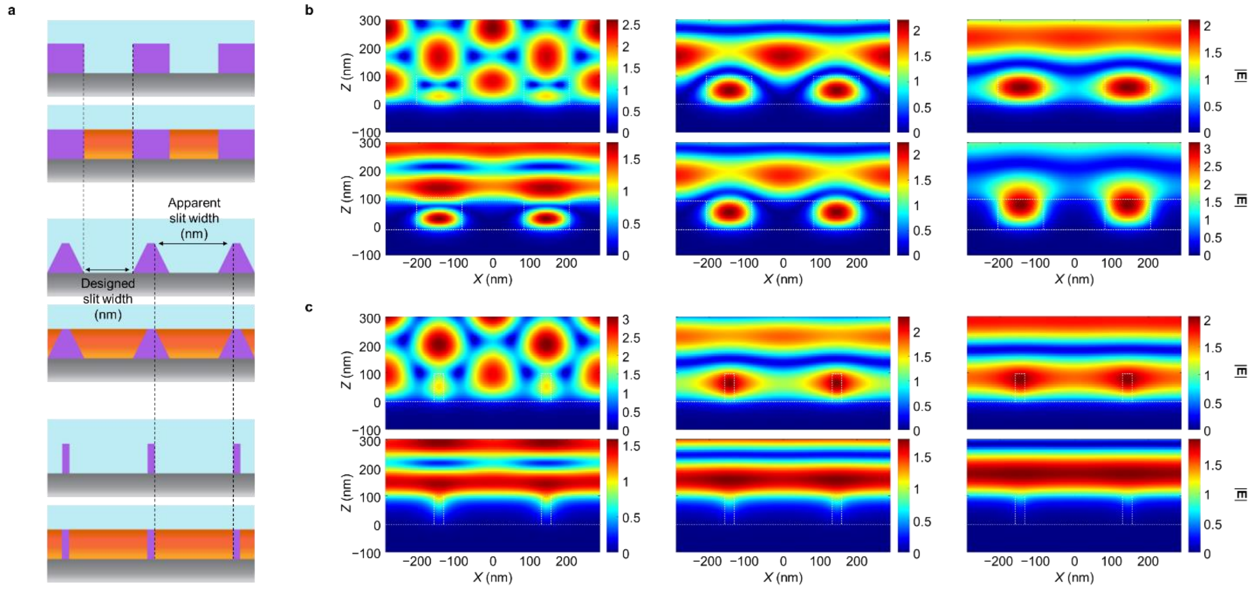

Supplementary Figure 17 | Electric field distributions in HCG with apparent slit width. a, Schematic of bare and Cu-filled HCG with p=290 nm, modelled with rectangular HCG bars and designed slit width of 165 nm (top); trapezoidal HCG bars (middle); and rectangular HCG bars and apparent slit width of 265 nm defined by the interspacing between the top part of the trapezoidal HCG bars (bottom). **b,c,** Electric field distributions of the bare (top) and Cu-filled (bottom) rectangular HCG with designed (**b**) and apparent (**c**) slit widths at wavelengths of 400 (left), 500 (middle), and 600 nm (right).

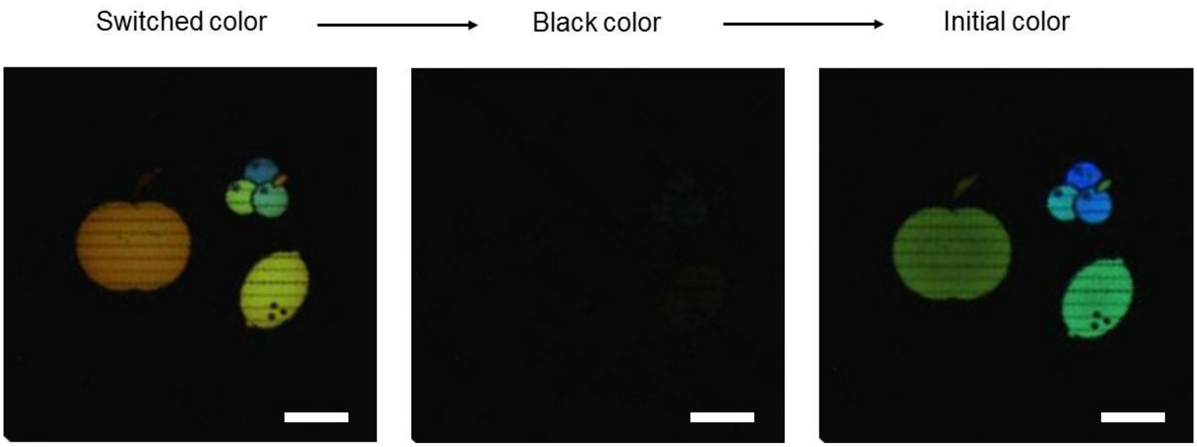

Supplementary Figure 18 | Turning off the fruit pixels. Cross-polarized OM images of fruit-shaped pixels with tuned (left), switched off (middle), and switched on (right) color. A potential of −1.9 V was applied for 3 s to completely fill the slits with Cu, after which 0.7 V was applied to create a porous Cu morphology. Pictures from left to right were taken at 1.0, 4.5, and 13.0 s.

Supplementary Note 8. Description of voltage controller

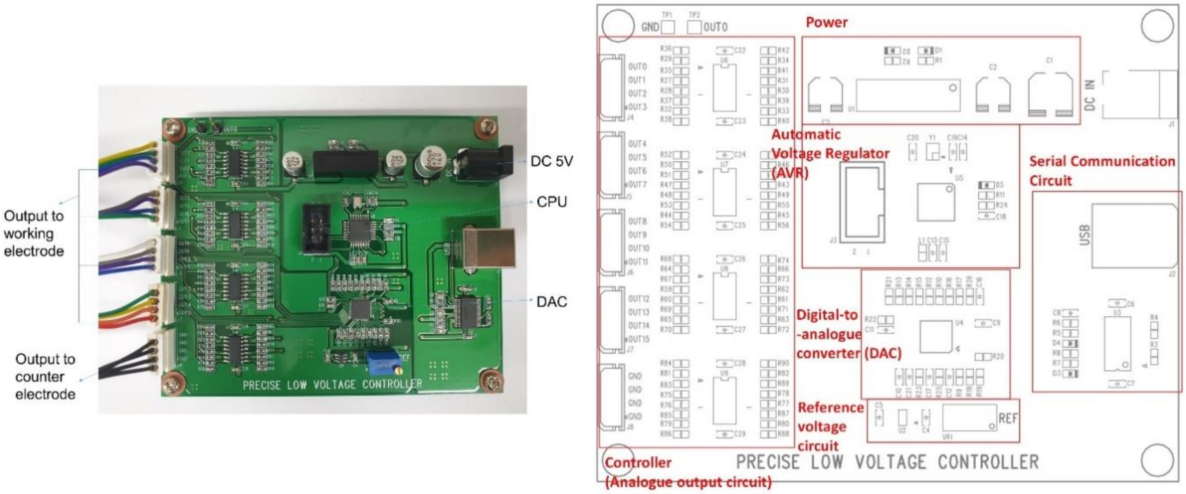

Supplementary Figure 19 | Blueprint of voltage controller. (left) Photo of PC board and (right) detailed blueprint showing the major components.

To control the 3×5 pixel matrix, a customized voltage controller was manufactured on a printed circuit board. The above photos show a picture of the voltage controller (left) and the corresponding blueprint (right). The controller was designed to precisely control the amplitude and time duration of bidirectional (+/−) DC voltage using a PC program. DC power (5V/1A) was supplied using an external adapter. The supplied voltage was adjusted to a setting-voltage using a multi-channel D/A converter and the impedance was reduced using a voltage buffer. The reference voltage was precisely adjusted using a trimmer. The controller was operated by a computer through a USB connection. Each pixel in the 3×5 pixel matrix was addressed by sourcing a potential through the respective WE output (colored wires) against the CE output (black wires). Detailed circuit diagrams of the components in the blueprint are displayed below.

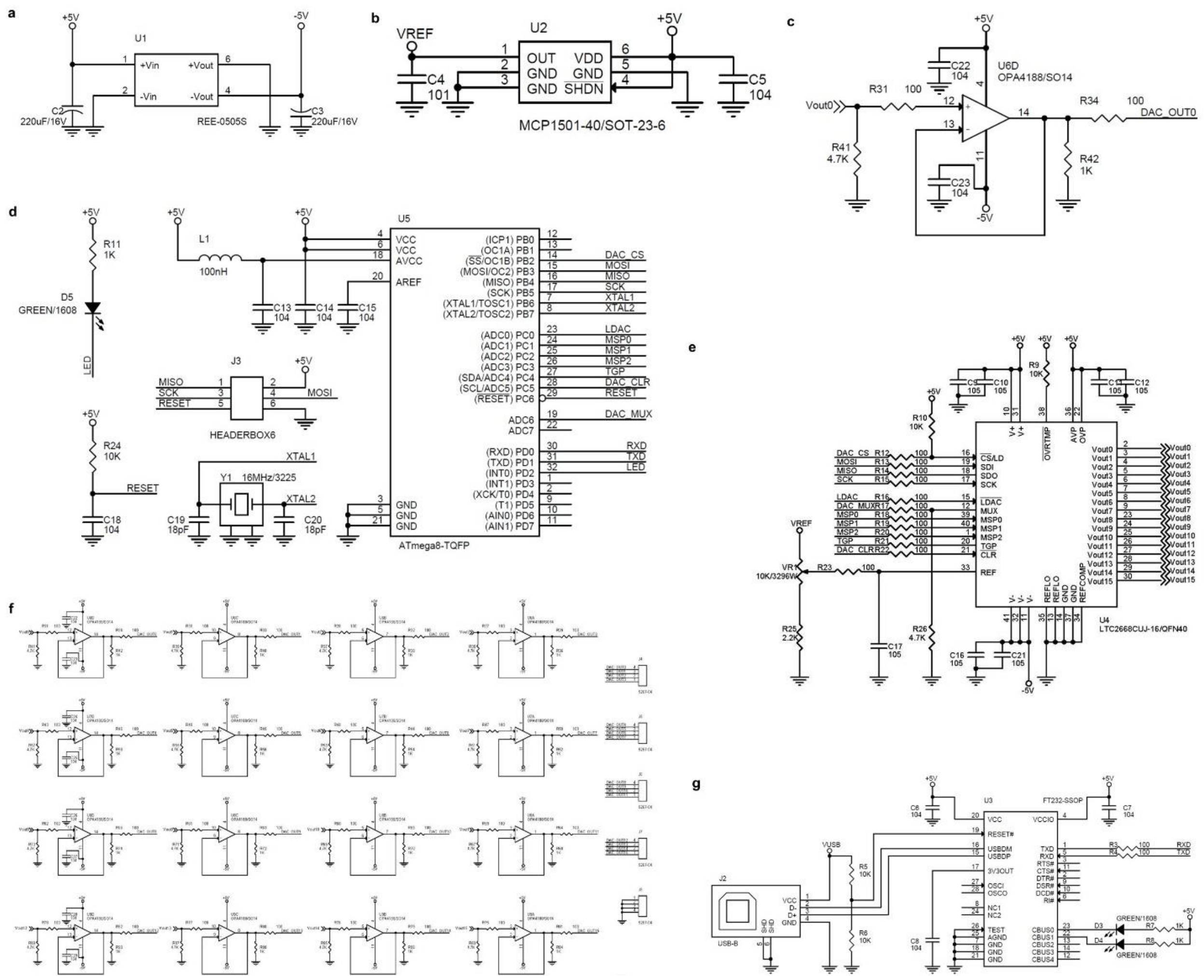

Supplementary Figure 20 | Circuit diagram of major components. Detailed diagrams of **a,** power unit, **b,** reference voltage unit, **c,** analogue output unit, **d,** automatic voltage regulator, **e,** digital-to-analogue converter (DAC), **f,** controller connections for analogue input to DAC output, and **g,** serial communication unit.

Components List

Component	Specification	PCB location
E/C	470uF/16V	C1
E/C	220uF/16V	C2,C3
C/C	101/1608	C4

C/C	104/1608	C5,C6,C7,C8,C13,C14,C15,C18,C22,C23,C24,C25,C26,C27,C28,C29
C/C	105/1608	C9,C10,C11,C12,C16,C17,C21
C/C	18pF/1608	C19,C20
LED	GREEN/1608	D1,D2,D3,D4,D5
CONNECTOR	DC-JACK-2MM	J1
CONNECTOR	USB-B	J2
CONNECTOR	HEADERBOX6	J3
CONNECTOR	5267_04	J4,J5,J6,J7,J8
INDUCTOR	100nH/1608	L1
RESISTOR	1K/1608	R1,R2,R7,R8,R11,R36,R38,R40,R42,R52,R54,R56,R58,R68,R70,R72,R74,R84,R86,R88,R90
RESISTOR	100/1608	R3,R4,R12,R13,R14,R15,R16,R17,R18,R19,R20,R21,R22,R23,R27,R28,R29,R30,R31,R32,R33,R34,R43,R44,R45,R46,R47,R48,R49,R50,R59,R60,R61,R62,R63,R64,R65,R66,R75,R76,R77,R78,R79,R80,R81,R82
RESISTOR	10K/1608	R5,R6,R9,R10,R24
RESISTOR	2.2K/1608	R25
RESISTOR	4.7K/1608	R26,R35,R37,R39,R41,R51,R53,R55,R57,R67,R69,R71,R73,R83,R85,R87,R89
IC	REE-0505S	U1
IC	MCP1501-40/SOT-23-6	U2
IC	FT232-SSOP	U3
IC	LTC2668CUJ-16/QFN40	U4
CPU	ATmega8-TQFP	U5
IC	OPA4188/SO14	U6,U7,U8,U9
TRIMMER	10K/3296W	VR1
CRYSTAL	16MHz/3225	Y1

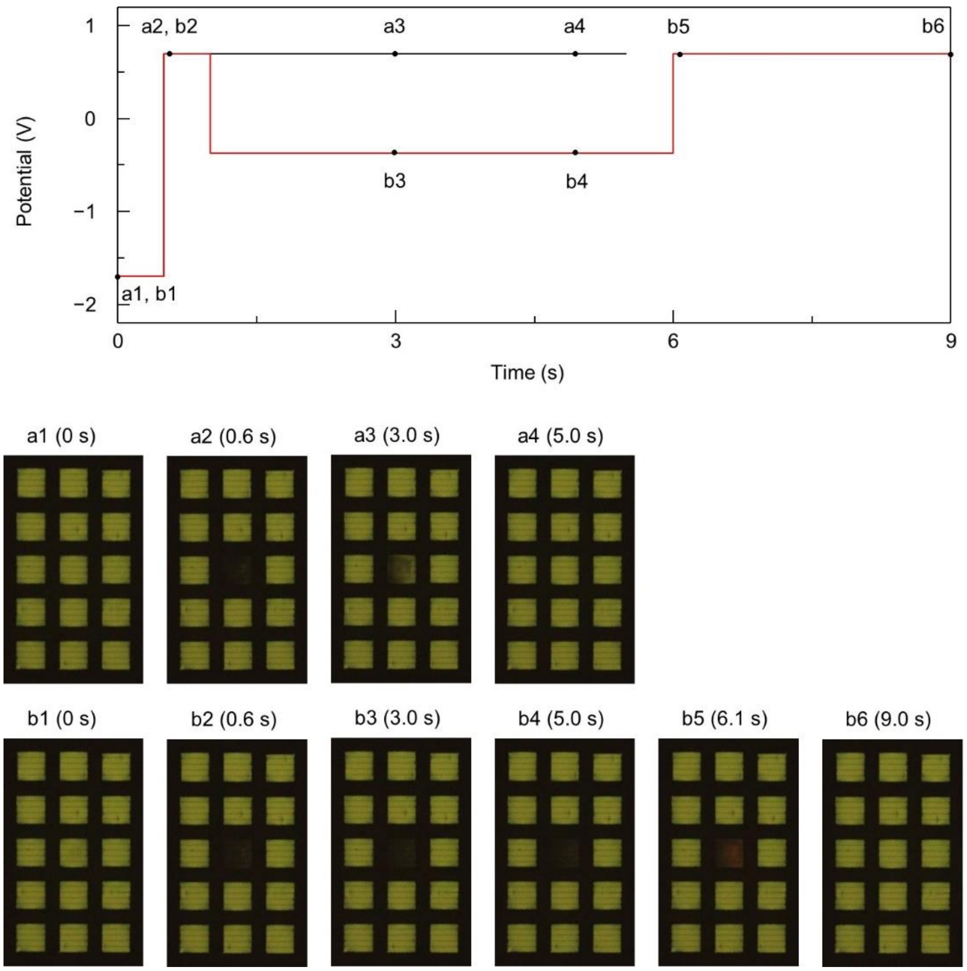

Supplementary Figure 21 | Maintaining black color in 3×5 pixel matrix. Temporal potential profiles without (a1~a4, black line) and with (b1~b6, red line) an offset potential of −0.37 V applied from 1 to 6 s to sustain the black color by cancelling the excess oxidative current (top). OM images of the respective pixel show the black color disappearing (middle) without the offset potential and remaining with it (bottom).

Supplementary References

1 Chang-Hasnain, C. J. & Yang, W. High-contrast gratings for integrated optoelectronics. *Adv. Opt. Photonics* **4**, 379-440 (2012).

2 Lochbihler, H. & Depine, R. Highly conducting wire gratings in the resonance region. *Appl. Opt.* **32**, 3459-3465 (1993).

3 Guillaumée, M., Dunbar, L. A. & Stanley, R. P. Description of the modes governing the optical transmission through metal gratings. *Opt. Express* **19**, 4740-4755 (2011).

4 Porto, J., Garcia-Vidal, F. & Pendry, J. Transmission resonances on metallic gratings with very narrow slits. *Phys. Rev. Lett.* **83**, 2845 (1999).

5 Garcia-Vidal, F. & Martin-Moreno, L. Transmission and focusing of light in one-dimensional periodically nanostructured metals. *Phys. Rev. B* **66**, 155412 (2002).

6 Skigin, D. C. & Depine, R. A. Transmission resonances of metallic compound gratings with subwavelength slits. *Phys. Rev. Lett.* **95**, 217402 (2005).

7 Sarkar, S., Lai, S. & Lemay, S. G. Unconventional electrochemistry in micro-/nanofluidic systems. *Micromachines* **7**, 81 (2016).

8 Małyszko, J. & Scendo, M. Electrode kinetics of the Cu (II)/Cu (I) system at platinum in water+ dimethylsulphoxide (DMSO) mixtures. *J. Electroanal. Chem. Interfacial Electrochem.* **250**, 61-72 (1988).

9 Haghi, A., Balköse, D. & Thomas, S. *Applied Physical Chemistry with Multidisciplinary Approaches*. (CRC Press, 2018).

10 Chen, L.-C. & Ho, K.-C. Interpretations of voltammograms in a typical two-electrode cell: application to complementary electrochromic systems. *Electrochim. Acta* **46**, 2159-2166 (2001).
