# Supplementary material for: Active electrochemical high-contrast gratings as on/off switchable and color tunable pixels
Source: Nat Commun. 2022 Jun 13;13:3391. doi: 10.1038/s41467-022-31083-z (PMC9192692; doi:10.1038/s41467-022-31083-z)
Supplement: Supplementary file 2 — Description of Additional Supplementary Files [file 41467_2022_31083_MOESM2_ESM.pdf]

Description of Additional Supplementary Files

File Name: Supplementary Movie 1
Description: Cross-polarized reflection of 12 pixels with periods from 290 to 510 nm undergoing electrochemical Cu deposition and dissolution within the HCG slits. Scale bar, 50 μm.

File Name: Supplementary Movie 2
Description: Cross-polarized reflection of fruit-shaped pixels undergoing electrochemical Cu deposition and dissolution within the HCG slits (color change). Scale bar, 90 μm.

File Name: Supplementary Movie 3
Description: Cross-polarized reflection of fruit-shaped pixels undergoing electrochemical Cu deposition and dissolution within the HCG slits (on-off switching). Scale bar, 90 μm.

File Name: Supplementary Movie 4
Description: Cross-polarized reflection of individual pixels in 3x5 matrix sequentially addressed. Scale bar, 50 μm.

File Name: Supplementary Movie 5
Description: Cross-polarized reflection of 3x5 pixel matrix displaying the word 'SURE'. Scale bar, 50 μm.

File Name: Supplementary Movie 6
Description: Cross-polarized reflection of 3x5 pixel matrix displaying the word 'NANO'. Scale bar, 50 μm.
